# Gradient Potassium Application Differentially Regulates Rhizosphere Bacterial and Fungal Communities in Cherry Tomato (*Solanum lycopersicum* var. *cerasiforme*)

**DOI:** 10.3390/plants15101545

**Published:** 2026-05-19

**Authors:** Chenpeng Gao, Guigui Wan, Ruifeng Cheng, Quansheng Li, Qinglu Fan, Jinxia Cui, Linnan Wu

**Affiliations:** 1 Department of Horticulture, College of Agriculture, Shihezi University, Shihezi 832003, China; 20232012045@stu.shzu.edu.cn (C.G.);; 2 Key Laboratory of Special Fruits & Vegetables Cultivation Physiology and Germplasm Resources Utilization of Xinjiang Production and Construction Corps, Department of Horticulture, College of Agriculture, Shihezi University, Shihezi 832003, China; 3 College of Life Sciences, Shihezi University, Shihezi 832003, China; 13150400690@163.com; 4 Institute of Farmland Water Conservancy and Soil Fertilizer, Xinjiang Academy of Agricultural and Reclamation Sciences, Shihezi 832000, China

**Keywords:** cherry tomato, potassium, rhizosphere microbiome, bacteria, fungi

## Abstract

Potassium (K) is an essential macronutrient for plants and plays a critical role in soil microbial processes. However, its systemic effects on rhizosphere microorganisms in high-value crops like cherry tomato remain poorly understood. This study established a potassium gradient (K0 represents the no-potassium application, K1 represents low-potassium application, K2 represents a moderate-potassium application, K3 represents the conventional-potassium application, and K4 represents excessive-potassium application) to investigate responses in growth and rhizosphere bacterial and fungal communities of cherry tomato. Moderate potassium (K2) significantly enhanced dry matter accumulation in cherry tomato. Bacterial and fungal communities displayed distinct patterns: bacterial structure shifted continuously along the gradient, with specific enrichment of functional genera (nitrogen-fixing *Ensifer*, biocontrol-related *Lysobacter*), increased unique OTUs, and gradual co-occurrence network optimization at K2. In contrast, fungal community composition and network structure showed threshold responses to potassium. Low K (K1) suppressed dominant *Ascomycota* and increased unclassified fungi, while high potassium (K4) enriched parasitic/pathogenic fungi (*Alternaria*, *Curvularia*), increased network modularity, and reduced stability. This microbial ecological perspective highlights that optimized potassium application regulates functional microorganisms and differentially shapes rhizosphere communities, providing a theoretical basis for precision potassium management in cherry tomato.

## 1. Introduction

Cherry tomato (*Solanum lycopersicum* var. *cerasiforme*), a high-value horticultural crop, is highly sensitive to potassium nutrition [1,2,3,4,5]. Adequate potassium supply plays a critical role in ensuring high yield, superior fruit quality, and desirable flavor development [6,7]. However, under intensive continuous-cropping systems, inappropriate potassium fertilizer management, whether deficient or excessive, not only reduces fertilizer use efficiency and increases production costs [8,9], but may also lead to secondary soil salinization [10], nutrient imbalance, and other soil degradation problems [11]. More importantly, the long-term deterioration of soil health, particularly the disruption of the rhizosphere microecosystem, can severely constrain the stable maintenance of plant health and fruit quality. Previous studies have shown that negative soil potassium balance is widespread in certain agricultural production regions of China [12]. Therefore, an in-depth understanding of how potassium management regulates cherry tomato health through its influence on rhizosphere microbial communities is of great practical significance for achieving sustainable and high-quality cherry tomato production.

Potassium (K), an essential macronutrient for plant growth, plays a central role in regulating cellular osmotic potential, maintaining ion homeostasis, and activating various enzymatic reactions [13,14,15,16,17,18]. Beyond its nutritional function, potassium also acts as a key regulator of the rhizosphere microenvironment by influencing rhizosphere physicochemical properties, root exudation, and microbial community composition [19,20,21,22]. Previous studies have demonstrated that potassium availability can significantly alter the secretion of amino acids and soluble sugars from wheat and mung bean roots [23], while also stimulating the release of organic acids from tea roots [24]. These root exudates not only directly affect soil pH and ionic balance [25], but also reshape rhizosphere microbial communities [26], thereby modifying the rhizosphere chemical environment. Furthermore, both potassium deficiency and different potassium application levels have been shown to significantly influence root exudation patterns. Collectively, these findings indicate that potassium is not only an essential plant nutrient but also a key driver of rhizosphere environmental regulation.

The rhizosphere represents a critical interface for plant–soil–microbe interactions, harboring highly diverse and functionally active bacterial and fungal communities [27,28]. These microorganisms form complex interaction networks and collectively drive key ecological processes, including soil organic matter decomposition, nutrient transformation, suppression of soil-borne pathogens, and regulation of plant growth [5,29,30,31]. Accordingly, the structure and function of rhizosphere microbial communities are widely recognized as key determinants of cherry tomato health and yield stability [32]. Rhizosphere microbial communities exhibit highly sensitive and functionally selective responses to plant-derived chemical signals. Among these, root exudates represent the primary carbon source input into the rhizosphere, and variation in their composition and flux is considered a central driver of microbial community assembly [33]. Differences in carbon utilization capacity and ecological strategies among microbial taxa result in directional shifts in community structure. For instance, increased organic acid exudation generally enhances the activity of microorganisms involved in mineral nutrient transformation, whereas labile carbon sources such as sugars preferentially stimulate the enrichment of fast-growing plant growth-promoting bacteria [25,34]. In addition, increasing evidence suggests that rhizosphere microbial community assembly is predominantly shaped by plant regulation and resource availability rather than stochastic processes [35]. Therefore, potassium-mediated regulation of root carbon allocation and rhizosphere chemical properties is likely to induce predictable microbial community restructuring and functional differentiation.

Beneficial microbial taxa selectively enriched under potassium application may exert significant downstream effects on plant health. These microorganisms not only promote plant growth by enhancing nutrient solubilization and uptake, but also improve plant defense capacity by inducing systemic resistance [36]. Potassium-solubilizing microorganisms (KSMs) convert approximately 90–98% of non-exchangeable soil potassium into plant-available forms through multiple mechanisms, including organic acid secretion (e.g., citric acid production), rhizosphere acidification, and chelation and ion-exchange processes, thereby facilitating nutrient acquisition and plant growth [37]. In addition, beneficial rhizosphere microorganisms can suppress the colonization of soil-borne pathogens via competitive exclusion and the production of antimicrobial compounds [38]. Collectively, these observations suggest that potassium application may promote plant growth by selectively enriching beneficial microbial taxa in the rhizosphere.

Although the regulatory effects of potassium on plant rhizosphere microbiomes have been reported, most existing studies have primarily focused on microbial responses under potassium deficiency or a single potassium application level. Consequently, there remains a lack of systematic understanding of rhizosphere microecological responses under a potassium gradient. In particular, within protected agricultural systems, knowledge regarding microbial community succession and ecological network dynamics driven by continuous potassium gradients is still limited. In this study, cherry tomato (*Solanum lycopersicum* var. *cerasiforme*) was used as the model crop. Five potassium (K_2_O) application levels were established: no potassium (K0, 0 kg ha^−1^ K_2_O), low potassium (K1, 150 kg ha^−1^ K_2_O), moderate potassium (K2, 225 kg ha^−1^ K_2_O), high potassium (K3, 300 kg ha^−1^ K_2_O), and excessive potassium (K4, 375 kg ha^−1^ K_2_O). We investigated the effects of a potassium gradient ranging from deficiency to excess on rhizosphere microbial communities. High-throughput amplicon sequencing combined with diversity analyses was employed to characterize microbial community structure. The objectives of this study were to address the following questions: (1) whether potassium supply exerts a linear or threshold effect on rhizosphere bacterial and fungal community dynamics; (2) which key bacterial and fungal taxa show specific enrichment or depletion along the potassium gradient; and (3) how potassium application levels influence rhizosphere microbial co-occurrence network structures. Overall, this study aims to systematically elucidate the regulatory effects of potassium levels on cherry tomato rhizosphere microbial communities from the perspectives of community composition, functional taxa, and ecological network interactions, thereby providing a theoretical basis for precision potassium management in cherry tomato production.

## 2. Results

### 2.1. Effects of Potassium Application Levels on the Growth of Cherry Tomato

**Figure 1:**
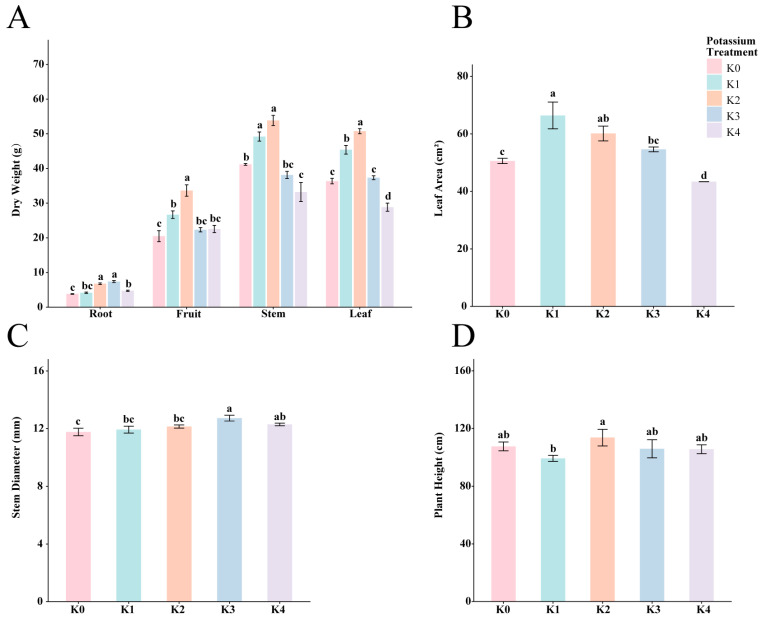
Effects of different potassium application levels on plant growth and dry matter accumulation. (**A**) Dry matter accumulation in plant organs (roots, fruits, stems, and leaves). (**B**) Leaf area. (**C**) Stem diameter. (**D**) Plant height. Data are presented as mean ± standard deviation (SD) (n=3). Different lowercase letters above the bars indicate significant differences among treatments at *p* < 0.05 based on one-way ANOVA followed by Tukey’s Honestly Significant Difference (HSD) test.

The effects of different potassium treatments on the growth and dry matter accumulation of cherry tomatoes are shown in Figure 1. Overall, with increasing potassium application rate, plant growth exhibited a trend of initial promotion followed by inhibition. The K2 treatment achieved the highest levels in stem, leaf, and fruit dry weights as well as plant height, which were significantly higher than those in other treatments. The K1 treatment significantly promoted leaf area expansion, but its promoting effect on dry matter was limited. Although the high-potassium treatments (K3 and K4) increased stem diameter to some extent, they significantly inhibited leaf area and dry matter accumulation. These results indicate that appropriate potassium application helps to coordinate the distribution and accumulation of assimilates, whereas excessive potassium application may induce growth inhibition.

### 2.2. Systematic Effects of Different Potassium Levels on Bacterial Diversity and Community Structure in Rhizosphere

#### 2.2.1. Potassium Application Alters Bacterial Diversity and Regulates OTU Composition

**Figure 2:**
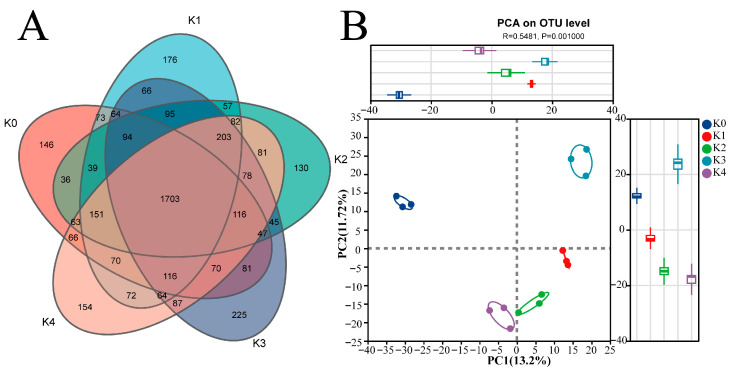
Effects of potassium application on the OTU composition and the community structure of rhizosphere bacteria. (**A**) Venn diagram at the OTU level. Numbers indicate shared or unique bacterial OTUs among treatments. (**B**) Principal coordinates analysis (PCoA) based on Bray–Curtis distance, illustrating differences in bacterial community structure among potassium treatments. Different colors represent different potassium treatments. Ellipses indicate 95% confidence intervals.

Venn diagram and principal coordinates analysis (PCoA) jointly indicated that potassium treatments significantly influenced the structure of the soil bacterial community (Figure 2). Venn diagram analysis showed that the numbers of detected bacterial OTUs in the five treatments (K0–K4) were 2935, 3125, 3020, 3154, and 3176. A total of 1703 OTUs were shared among all treatments. The numbers of unique OTUs in each treatment (K0–K4) were 146, 176, 130, 225, and 154 (Figure 2A). Overall, both the total number of OTUs and the number of treatment-specific OTUs in the potassium-treated groups were higher than those in the control (K0). Specifically, K0-unique OTUs belonged mainly to Patescibacteria and Chloroflexota; K1–K2-unique OTUs included *Ensifer* and *Lysobacter*; and K3–K4-unique OTUs were primarily affiliated with *Bacillus*, *Arthrobacter*, *Oceanobacillus*, and *Pseudomonas*. PCoA based on Bray–Curtis distances showed that bacterial community composition shifted in response to potassium treatments. PERMANOVA confirmed a significant effect of potassium application (R^2^ = 0.5481, *p* = 0.001), indicating that the overall bacterial community structure differed significantly among treatments.

#### 2.2.2. Phylum- and Genus-Level Bacterial Community Responses to Potassium Gradients

**Figure 3:**
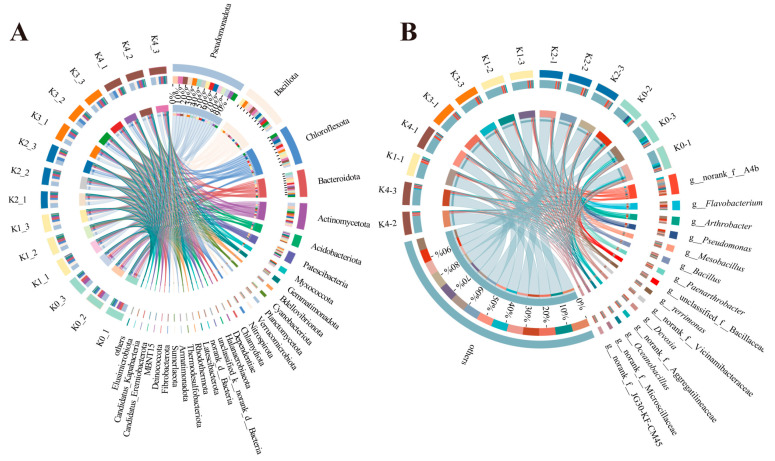
Effects of potassium application on the composition of rhizosphere bacterial communities at different taxonomic levels. (**A**) Relative abundance of dominant bacterial phyla under different potassium application levels. (**B**) Relative abundance of dominant bacterial genera under different potassium application levels.

At the phyla level (Figure 3A; Table A1), rhizosphere bacterial communities under all potassium treatments were dominated by Pseudomonadota, Actinomycetota, Bacillota, Chloroflexota, and Acidobacteriota. Compared with the K0 control, K1 (low potassium) significantly increased the relative abundance of Actinomycetota (24.27%), while K4 (high potassium) showed the highest proportion of Pseudomonadota across all treatments (32.59%). In K3 (moderate–high potassium), Bacillota exhibited the highest relative abundance (17.85%). At the genus level (Figure 3B; Table A2), potassium application selectively enriched bacterial genera with distinct ecological functions. K1 and K2 (low to moderate–low potassium) promoted the enrichment of nitrogen-fixing genera, including *Ensifer* (average relative abundances: 1.01% and 1.89%), and biocontrol-related genera such as *Lysobacter* (1.04% in K2). K3 significantly enriched several halotolerant or halophilic genera, including *Gracilibacillus* (1.76%) and *Oceanobacillus* (1.84%). In K4, the organic matter-degrading genus *Planifilum* reached the highest relative abundance (1.69%). These results indicate that potassium application not only altered the overall structure of the rhizosphere bacterial communities but also selectively enriched taxa with functional traits, including nitrogen fixation, biocontrol, salt tolerance, and organic matter decomposition.

#### 2.2.3. Identification of Differentially Abundant Genera and Implications for Community Succession Dynamics

**Figure 4:**
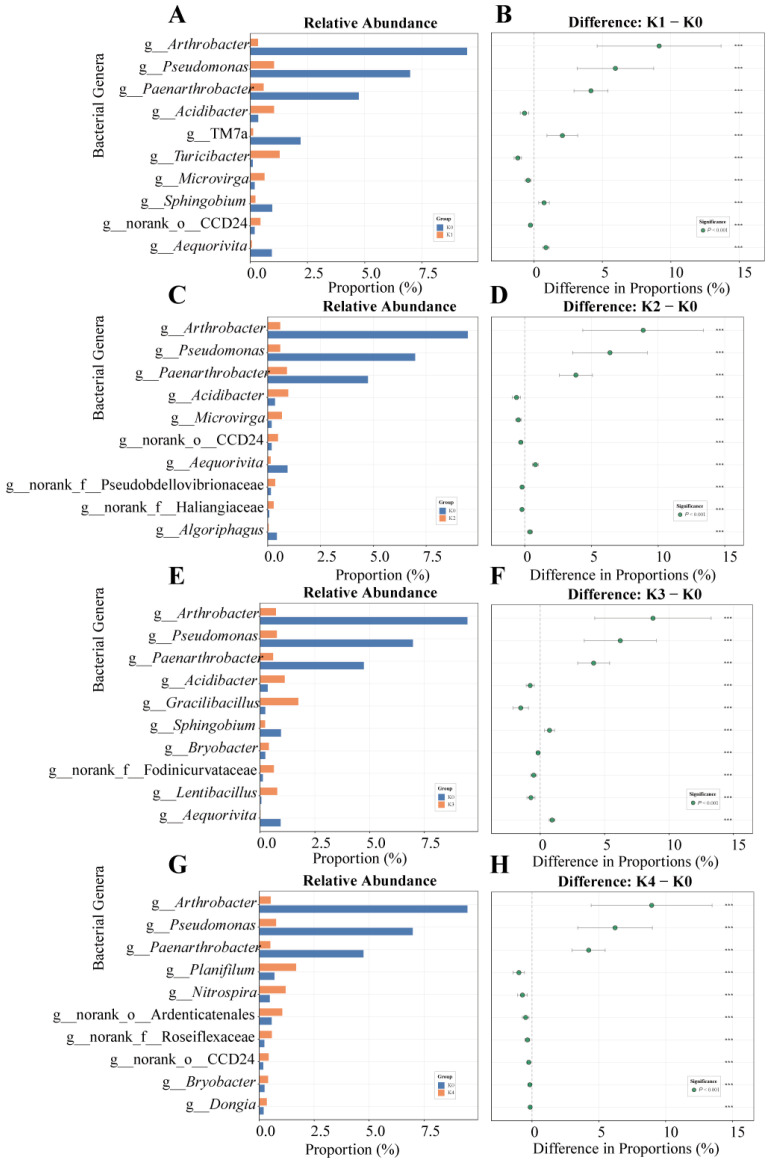
Identification of differentially abundant bacterial genera under different potassium treatments. This figure shows the relative abundance and effect sizes of bacterial genera that differed significantly between each potassium treatment (K1–K4) and the control (K0). Panels (**A**,**C**,**E**,**G**) show the average relative abundances of the top 10 significantly different bacterial genera in each treatment group (bar plots, mean ± SD, *n* = 3). Panels (**B**,**D**,**F**,**H**) show the mean differences in abundances between treatment and control groups (forest plots, mean difference ± 95% confidence interval). Note: differential taxa were identified based on one-way ANOVA followed by Tukey’s HSD test; *p* < 0.05; *** *p* < 0.001.

Using Tukey’s HSD test for multiple comparisons, a total of 224 bacterial genera with significant differences among potassium treatments was identified. Among these, 28 core genera consistently showed significant responses across all treatments (Figure 4). Notably, *Arthrobacter*, *Pseudomonas*, and *Paenarthrobacter* exhibited the widest response range and the largest effect sizes (e.g., effect value of *Arthrobacter* up to 9.145). Their rapid proliferation during the mid-to-late stages of treatment indicated a functional reassembly phase in the community. In contrast, *Nitrospira* and the Acidobacteriota genera *Bryobacter* and *Acidibacter* were consistently suppressed across all potassium treatments. During the early succession stage (K1 treatment), transient enrichments of *Clostridium* and *Romboutsia* indicated initial community disturbances induced by potassium application. At the highest potassium level (K4), an unclassified genus within the family Saccharimonadaceae (*norank_f__Saccharimonadaceae*) became significantly enriched, suggesting the formation of complex interspecies interactions.

#### 2.2.4. LEfSe Analysis Reveals the Key Microbial Groups Responding to the Potassium Gradient

**Figure 5:**
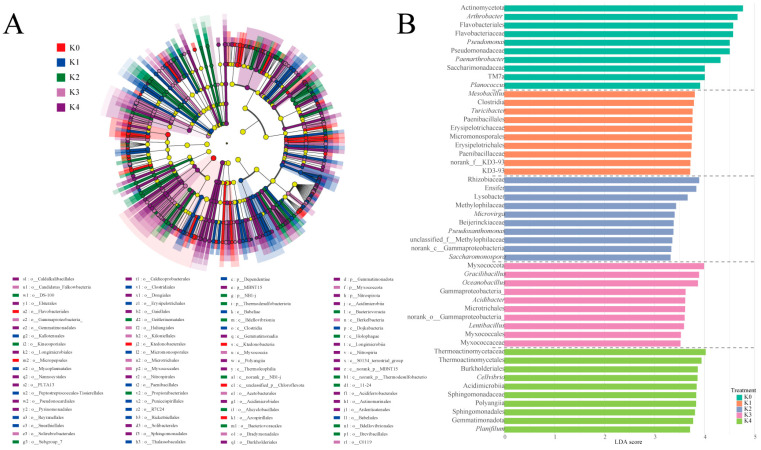
LEfSe analysis showing differences in rhizosphere microbial communities across potassium treatments. (**A**) Phylogenetic tree showing microbial taxa significantly enriched under each potassium treatment; (**B**) LDA score bar plot indicating dominant taxa with significant differences across treatments. The LDA score represents the effect size estimated by linear discriminant analysis, reflecting the contribution of each taxon to differences among treatments (LDA score > 3.0). The grey dashed lines separate the taxa enriched under different potassium treatments.

LEfSe analysis (Figure 5) suggested that potassium gradients were associated with shifts in rhizosphere bacterial community composition. Under potassium deficiency (K0), taxa related to stress tolerance and metabolite dependence, including Patescibacteria (*Saccharimonadia*), *Sphingomonas*, *Pseudomonas*, and *Arthrobacter*, showed relatively higher enrichment. With mild potassium application (K1), the bacterial community tended to be associated with taxa involved in organic matter transformation and microbial interactions, including *Erysipelotrichaceae*, *Mycoplasmatales*, *Dependentiae*, and *Myxococcales*. Under moderate–low potassium (K2), decomposition- and biocontrol-related taxa such as *Lysobacter* and *Massilia* showed higher relative enrichment. In the moderate–high-potassium treatment (K3), *Cellvibrio* and *Rhodanobacter* were relatively more abundant. Under high potassium (K4), taxa including *Bacillus*, *Nitrospira*, and *Burkholderiales* exhibited higher enrichment patterns.

#### 2.2.5. Nonlinear Responses of Bacterial Co-Occurrence Network Structure to Potassium Application Levels

**Figure 6:**
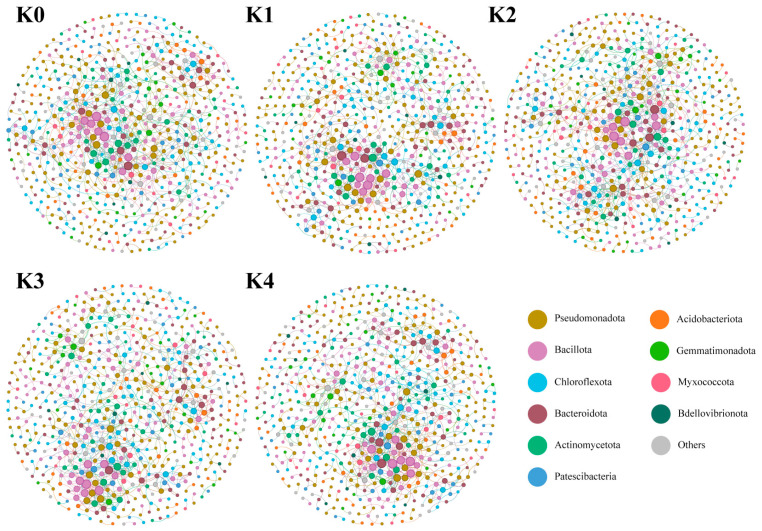
Bacterial co-occurrence networks in the rhizosphere of cherry tomato under different potassium treatments. The network illustrates the interactions among microbial taxa at the OTU level. Each node represents a specific microbial taxon, and the size of the node is proportional to its relative abundance (or degree centrality). Nodes are colored according to their taxonomic assignments at the phylum level, with the top 10 dominant phyla and their relative percentages shown in the upper legend. The edges (connections) between nodes indicate significant positive or negative correlations between taxa (e.g., Spearman’s |r| > 0.8, *p* < 0.05).

Based on the OTU level, the network topology parameters for each potassium treatment revealed that the bacterial network exhibited high structural stability across the potassium gradient: the number of nodes (621–628), clustering coefficient (~0.32), and modularity (~0.80) remained highly consistent among treatments (Figure 6; Table A3). Network complexity did not increase linearly with potassium application rate; instead, it reached an optimum under the K2 treatment, where the number of edges (943) and the average degree (3.017) were the highest among all treatments. This pattern suggests that an appropriate level of potassium input may promote tighter bacterial co-occurrence relationships, whereas potassium deficiency or excess may lead to a moderate simplification of network connectivity.

### 2.3. Reshaping Effects of Different Potassium Levels on Rhizosphere Fungal Diversity and Community Structure

#### 2.3.1. Threshold Responses of Fungal OTU Composition and Overall Community Structure to Potassium Application

**Figure 7:**
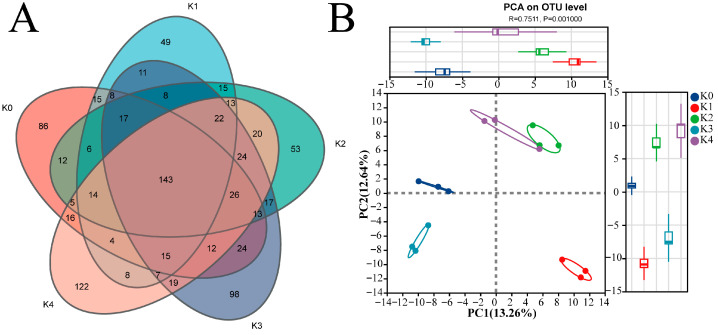
Effects of potassium application on fungal OTU composition and community structure in tomato rhizosphere. (**A**) Venn diagram at the OTU level. Numbers indicate shared or unique fungal OTUs among treatments. (**B**) Principal coordinates analysis (PCoA) based on Bray–Curtis distances. Different colors represent different potassium treatments. Ellipses represent 95% confidence intervals.

As shown in Figure 7, Venn diagram and PCoA analyses demonstrated that potassium application significantly altered both the OTU composition and overall structure of the tomato rhizosphere fungal community. Although a core set of OTUs was shared across all treatments, substantial differences were observed in the number of treatment-specific OTUs. Venn diagram analysis revealed that the numbers of fungal OTUs detected across the five potassium treatments (K0–K4) were 416, 355, 408, 464, and 470, respectively. A total of 143 core OTUs were shared among all treatments. The numbers of treatment-specific OTUs varied among treatments: K0 contained 86 unique OTUs; K1 had 49; K2 had 53; while K3 and K4 showed higher values, with 98 and 122 unique OTUs, respectively, with K4 exhibiting the highest number. K0-unique OTUs encompassed diverse functional groups; K1-unique OTUs were predominantly unclassified fungi; K2-unique OTUs included core dominant genera such as *Chordomyces*; K3-unique OTUs were enriched in saprotrophic Mortierellomycota; and K4-unique OTUs were mainly affiliated with potentially pathogenic genera such as *Curvularia* and *Alternaria*. PCoA based on Bray–Curtis distances showed that fungal community composition shifted in response to potassium treatments. PERMANOVA confirmed a significant effect of potassium application (R^2^ = 0.7511, *p* = 0.001), indicating that the overall fungal community structure differed significantly among treatments.

#### 2.3.2. Pronounced Reconstruction of Fungal Community Composition at the Phylum and Genus Levels

**Figure 8:**
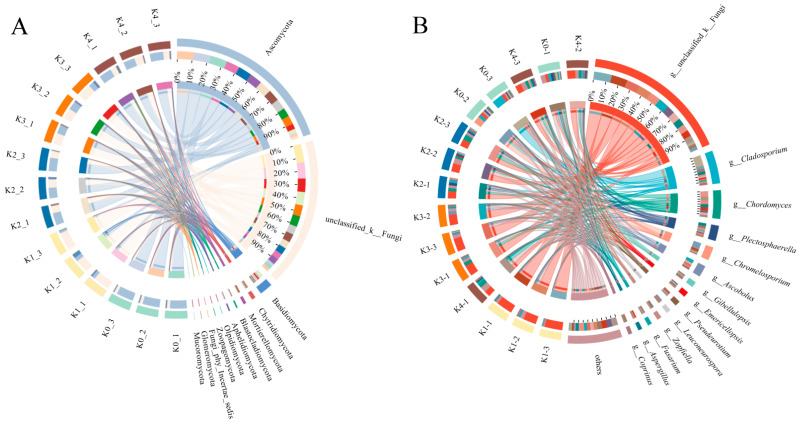
Effects of potassium application on the composition of rhizosphere fungal communities at different taxonomic levels. (**A**) Relative abundance of dominant fungal phyla under different potassium application levels. (**B**) Relative abundance of dominant fungal genera under different potassium application levels.

As shown in Figure 8A, phylum-level analysis revealed that potassium application profoundly reshaped soil fungal community structure. Under the no-potassium treatment (K0), *Ascomycota* overwhelmingly dominated the fungal community. Under low-potassium input (K1), the relative abundance of *Ascomycota* sharply declined, accompanied by a pronounced increase in unclassified fungi. With increasing potassium application to moderate and high levels (K2–K4), community structure gradually recovered: the relative abundance of *Ascomycota* rebounded to approximately 50–60%, while unclassified fungi decreased. Meanwhile, *Chytridiomycota* and *Blastocladiomycota* showed slight increases under high-potassium conditions. Overall, the fungal community composition exhibited a trajectory characterized by initial suppression, subsequent recovery, and eventual promotion along the potassium gradient. Genus-level analysis (Figure 8B) further indicated substantial compositional shifts. Under K0, the community was dominated by *Cladosporium* and *Gibellulopsis*, whereas *Chordomyces* became the most prominent genus under K4. In addition, the relative abundances of *Aspergillus* and *Emericellopsis* increased progressively with rising potassium levels. Several key functional genera displayed distinct responses to potassium availability: *Fusarium* exhibited broad tolerance across treatments, while *Trichoderma* was relatively more abundant under low-potassium conditions. Collectively, these results suggest that potassium application regulates fungal community structure and functional group dynamics by altering soil physicochemical conditions.

#### 2.3.3. LEfSe Analysis Reveals the Key Fungal Groups Responding to the Potassium Gradient

**Figure 9:**
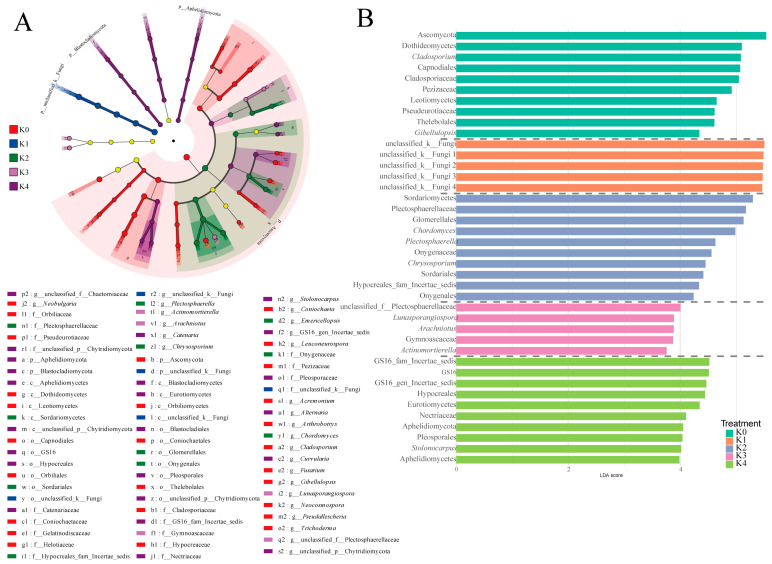
LEfSe analysis of significantly enriched microbial taxa in the cherry tomato rhizosphere under different potassium treatments. (**A**) Phylogenetic tree illustrating microbial taxa significantly enriched under each potassium treatment; (**B**) LDA score bar plot showing dominant taxa with significant differences among treatments (LDA score > 3.0). The grey dashed lines separate the taxa enriched under different potassium treatments.

LEfSe analysis demonstrated that potassium application markedly shaped the distribution of fungal biomarkers in the tomato rhizosphere (Figure 9). Under the K0 treatment, the fungal community exhibited high complexity, with biomarkers spanning from phylum to genus levels, including *Ascomycota* and representative genera such as *Alternaria*, *Fusarium*, and *Trichoderma*. Under low-potassium conditions (K1), unclassified fungi were significantly enriched, indicating the dominance of previously unresolved or unknown taxa. At the moderate potassium level (K2), *Chordomyces* and *Plectosphaerella* emerged as core biomarkers, reflecting a shift in dominant taxa. Under high-potassium input (K3), saprotrophic fungal groups affiliated with *Mortierellomycota*, including *Lunasporangiospora* and *Actinomortierella*, were significantly enriched. At the highest potassium level (K4), parasitic and pathogenic fungi became dominant, including taxa affiliated with *Aphelidiomycota* and *Blastocladiomycota*, as well as genera such as *Alternaria* and *Curvularia*. Overall, along the increasing potassium gradient, fungal biomarkers exhibited a clear successional pattern characterized by a transition from “functionally diverse” to “unknown stress-associated”, followed by “core dominant”, “saprotrophic”, and finally “parasitic/pathogenic” assemblages. These results indicate that potassium application is a key driver regulating fungal community structure and functional reorganization in the rhizosphere.

#### 2.3.4. Response Patterns of Fungal Co-Occurrence Networks to Potassium Application Levels

**Figure 10:**
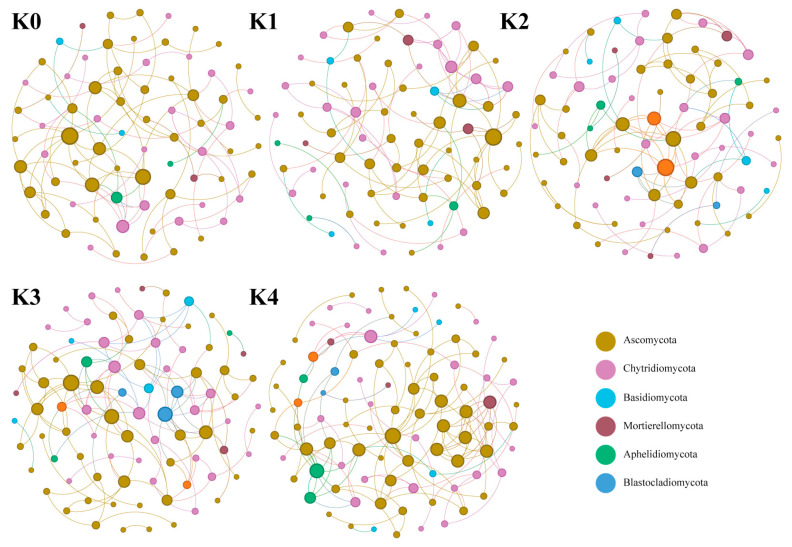
Fungal co-occurrence networks in the cherry tomato rhizosphere under different potassium treatments. The network illustrates the interactions among fungal taxa at the OTU level. Each node represents a specific fungal taxon, and the size of the node is proportional to its relative abundance (or degree centrality). Nodes are colored according to their taxonomic assignments at the phylum level, with the top six dominant fungal phyla (including Ascomycota, Chytridiomycota, Basidiomycota, etc.) and their relative percentages shown in the upper legend. The edges (connections) between nodes indicate significant positive or negative correlations between taxa (e.g., Spearman’s |r| > 0.8, *p* < 0.05).

Unlike the stable bacterial network, the fungal network showed distinct responses (Figure 10; Table A4). Under K0–K2 treatments, the network remained small, with similar parameters across conditions (approximately 83 nodes). However, when the potassium application rate reached K3 and K4, the network topology changed dramatically: the number of nodes increased (reaching 99 under K4), and the number of edges rose to 126. The clustering coefficient (0.571) and modularity (0.829) peaked under the K4 treatment, suggesting that high-potassium conditions may favor the formation of a more aggregated and modular fungal co-occurrence structure.

## 3. Discussion

### 3.1. Potassium Application Significantly Affected Cherry Tomato Growth

This study revealed that the response of cherry tomato growth to potassium application levels followed a “low promotion–optimal–high inhibition” pattern. Plant height and the dry weights of stems, leaves, and fruits were significantly higher under the K2 treatment than under the other treatments, indicating that appropriate potassium application effectively promotes dry matter accumulation and vegetative growth. This finding is consistent with the physiological roles of potassium in plants. Under adequate supply conditions, potassium functions as a key osmotic regulator that facilitates stomatal movement, enhances photosynthetic efficiency, and improves assimilate transport. In addition, potassium activates multiple enzyme systems involved in carbon assimilation and metabolism, thereby promoting the conversion of structural and storage compounds and ultimately increasing dry matter accumulation [18,39].

In contrast, although leaf area increased significantly under low-potassium treatment (K1), dry matter accumulation remained limited. This is consistent with previous studies showing that potassium deficiency restricts the transport and utilization of assimilates, thereby limiting their conversion into dry matter [7,40]. Under high-potassium treatments (K3–K4), both leaf area and dry matter accumulation were significantly reduced, indicating that excessive potassium application exerts an inhibitory effect on plant growth. Overall, cherry tomato growth exhibited a clear threshold response to potassium application, with K2 representing the optimal level. Deviation from this optimal range, either below or above, resulted in a decline in growth performance.

### 3.2. Continuous Response of Bacterial Community Under a Potassium Gradient

In the analysis of OTU composition, diversity ordination, and differential abundance, the bacterial community exhibited a continuous response pattern along the potassium gradient (Figure 2, Figure 3 and Figure 4). Under low-to-moderate potassium inputs (K1–K2), the relative abundances of *Ensifer* and *Lysobacter* were higher. Notably, *Ensifer* includes taxa associated with nitrogen cycling processes, while certain *Lysobacter* members are known to produce antimicrobial metabolites and extracellular hydrolases [41,42], suggesting potential roles in nutrient transformation and pathogen suppression. In contrast, under high-potassium treatment (K4), the relative abundances of *Arthrobacter* and *Pseudomonas* increased. These taxa are widely recognized for their strong environmental adaptability and metabolic versatility, which may confer competitive advantages under altered soil conditions [43]. Meanwhile, the relative abundance of the environmentally sensitive genus *Nitrospira* continuously declined along the potassium gradient, indicating that potassium-induced changes in rhizosphere conditions may negatively affect nitrifying microbial populations and potentially disrupt nitrification-related processes [44].

LEfSe analysis further identified key bacterial taxa that were differentially enriched under distinct potassium levels. Under potassium-deficient conditions (K0), Patescibacteria was significantly enriched. This phylum belongs to the Candidate Phyla Radiation (CPR) group, whose representative genomes are typically highly reduced, lacking complete biosynthetic pathways and relying on metabolic interactions with other microorganisms for essential metabolites. Therefore, its enrichment is often considered indicative of resource-limited or oligotrophic environmental conditions [45,46]. Under low-to-moderate-potassium treatments (K1–K2), *Ensifer* and *Lysobacter* maintained relatively high abundances, consistent with their potential roles in nutrient cycling and microbial interaction networks. In contrast, *Bacillus* was significantly enriched under high-potassium conditions (K4). Members of the genus *Bacillus* are well-known for their ability to form endospores and exhibit strong tolerance to osmotic stress, salinity, and variations in ionic strength [47]. Therefore, the enrichment of *Bacillus* may be associated with potassium-induced changes in rhizosphere physicochemical conditions.

Overall, the bacterial community exhibited a continuous compositional shift along the potassium gradient. Under potassium-deficient conditions, oligotrophic and resource-limited taxa were predominantly enriched. Under optimal potassium application (K2), beneficial and functionally active microbial groups were more abundant. In contrast, high-potassium conditions favored the enrichment of stress-tolerant and metabolically flexible taxa, indicating a gradual transition in community assembly patterns with increasing potassium supply. This compositional shift was closely aligned with plant growth responses under different potassium levels. Specifically, the highest relative abundance of beneficial microbial groups corresponded to optimal plant growth under K2, whereas the dominance of stress-tolerant taxa under K4 coincided with growth inhibition. These results suggest a coordinated response between plant performance and rhizosphere microbial community assembly along the potassium gradient.

### 3.3. Threshold Response of Fungal Community Under a Potassium Gradient

This study also revealed a threshold response pattern of fungal communities along the potassium gradient. Under low-potassium stress (K1), the relative abundance of core fungal taxa, particularly members of Ascomycota, decreased in the rhizosphere of cherry tomato, whereas the proportion of unclassified fungal taxa increased. As potassium supply increased to moderate and high levels (K2–K4), the fungal community shifted toward a more stable state, characterized by a recovery in the abundance of Ascomycota and a corresponding decline in unclassified taxa (Figure 8A). Ascomycota represents a key functional group involved in the decomposition of plant-derived polysaccharides in soil [48]. The pronounced decline in Ascomycota under K1 suggests a potential weakening of carbon decomposition capacity under potassium deficiency, which may be associated with reduced carbon input from root exudates. This shift is consistent with the observed phenotype, where increased leaf area was accompanied by limited dry matter accumulation under low-potassium conditions.

LEfSe analysis further revealed a sequential turnover of differential fungal biomarkers across potassium treatments (Figure 9). Under K0 treatment, indicator taxa spanned multiple taxonomic levels from phylum to genus, including Ascomycota, *Alternaria*, *Fusarium*, and *Trichoderma*, suggesting a relatively diverse and functionally heterogeneous fungal community under potassium deficiency. Under K1, unclassified fungal taxa dominated the indicator spectrum. Although their taxonomic identities remain unresolved, their enrichment likely reflects adaptive restructuring of fungal communities under potassium limitation. Previous studies have suggested that certain fungi may contribute to enhancing plant nutrient acquisition under nutrient-stressed conditions, potentially facilitating survival in low-potassium environments [49]. Thus, the enrichment of unclassified taxa may represent a selective response of the community to potassium deficiency. At K2, *Chordomyces* and *Plectosphaerella* emerged as core indicator taxa, suggesting a shift toward a more stable community structure with altered dominant functional groups. Under K3, taxa affiliated with Mortierellomycota, including *Lunasporangiospora* and *Actinomortierella*, were enriched, which are primarily recognized as saprophytic organisms involved in organic matter decomposition. In contrast, K4 was characterized by the dominance of Aphelidiomycota, Blastocladiomycota, *Alternaria*, and *Curvularia*. Notably, *Alternaria* and *Curvularia* have been reported to include species associated with plant diseases such as tomato leaf spot [50,51]. Overall, the indicator taxa exhibited a clear successional trajectory along the potassium gradient, transitioning from functionally diverse communities (K0) to unclassified adaptive assemblages (K1), then to stable core taxa (K2), followed by saprophytic-dominated communities (K3), and finally to pathogen-associated assemblages under high-potassium conditions (K4). This nonlinear shift indicates that fungal community assembly is strongly threshold-dependent, and that stable rhizosphere fungal structure requires potassium levels to exceed a critical ecological threshold. Below this threshold, substantial restructuring of community composition occurs.

Bacterial and fungal communities exhibited distinct response patterns to the potassium gradient: continuous in bacteria but threshold-like in fungi. Nevertheless, both showed coordinated changes with plant growth status across treatments. Plant growth was optimal under K2, coinciding with a stable fungal community structure characterized by core indicator taxa such as *Chordomyces* and *Plectosphaerella*. In contrast, under K4, where plant growth was inhibited, fungal indicator taxa shifted toward assemblages containing potential plant-associated pathogens, including *Alternaria* and *Curvularia*. These results suggest that high-potassium conditions may drive rhizosphere fungal niche reorganization toward assemblages more favorable to saprophytic and potentially pathogenic taxa. More broadly, potassium-driven shifts in microbial community composition were closely coupled with plant performance, indicating a strong plant–microbiome co-response along the potassium gradient. Although functional attributes of these microbial groups have been individually reported, their ecological roles and the stability of these differential assemblages within the rhizosphere ecosystem remain to be further validated.

### 3.4. Potassium Gradient Regulates the Differential Co-Occurrence Network Structures of Rhizosphere Bacteria and Fungi

Potassium application not only altered the composition of rhizosphere microbial communities, but also reshaped the potential interaction networks between bacteria and fungi. However, bacteria and fungi exhibited fundamentally different responses in network structure under the potassium gradient. The bacterial co-occurrence network remained relatively stable across K0–K4 treatments, with only minor variations in the number of nodes and modularity, indicating strong structural robustness. Dominant bacterial phyla, including Pseudomonadota, Bacillota, Chloroflexota, and Bacteroidota, consistently occupied central positions across multiple network modules. Previous studies have demonstrated that major bacterial lineages such as Proteobacteria and Actinobacteria often maintain stable dominance across diverse agricultural management practices and environmental conditions, forming the core framework of rhizosphere microbial communities and playing key roles in maintaining community structural stability and ecological functioning [52]. From a topological perspective, network complexity (e.g., number of edges and average degree) peaked under the K2 treatment, which also corresponded to the highest aboveground dry matter. This suggests that optimal potassium supply promotes tighter and more coordinated interactions among bacterial core taxa, potentially enhancing community-level functional integration. The observed stability of the bacterial network may be attributed to their relatively short life cycles and flexible metabolic strategies (r-strategy), enabling rapid adjustment of interspecific interactions in response to potassium gradients without causing large-scale structural reorganization [43].

In contrast to bacteria, the fungal co-occurrence network exhibited a distinct two-phase response along the potassium gradient. During the K0–K2 stage, the fungal network, with Ascomycota-dominated backbone taxa, maintained relatively low complexity. However, when potassium application increased to K3–K4, both the number of nodes and edges increased markedly, and network clustering coefficient and modularity reached their maximum under K4 treatment. These results suggest that high-potassium input imposes stronger environmental filtering on fungal communities, driving a transition from a loosely connected network to a highly modularized structure. Similar patterns have been reported in fungal networks under extreme environmental stresses such as drought [53,54], where communities reorganize into tightly connected functional modules as a strategy to enhance stress resistance. In this study, the pronounced modular reorganization under high-potassium conditions may indicate that potassium-induced environmental filtering exceeds the buffering capacity of the fungal community, promoting the formation of stress-adapted and functionally clustered modules. These modules may include taxa associated with stress tolerance and potential plant–pathogenic interactions. Notably, this network-level shift is consistent with the observed reduction in plant growth and the increased abundance of pathogen-associated fungal taxa under K4 treatment. However, the causal relationships underlying these patterns require further experimental validation.

Overall, there are fundamental differences in the response strategies of bacterial and fungal networks to potassium application: bacterial networks maintained structural stability with flexible metabolic strategies and achieved optimal connectivity under appropriate potassium application levels; in contrast, the fungal network underwent pronounced modular reorganization under high-potassium stress. This difference reflects the intrinsic distinctions between the two microbial groups in niche occupancy and physiological and metabolic characteristics—bacteria have short life cycles and respond rapidly to nutrient fluctuations, whereas fungi possess more complex nutrient acquisition mechanisms and higher carbon demands, making them more sensitive to environmental stress [55]. The above network structural changes were consistent with plant growth performance across treatments: plant growth was optimal under K2 treatment, and bacterial network complexity also reached its peak; under K4 treatment, plant growth was inhibited, and the fungal network underwent significant modular reorganization. However, the stability of the bacterial network and the threshold response pattern of the fungal network observed in this study should be considered preliminary inferences regarding potassium-driven rhizosphere microecological succession. Given that each treatment included only three biological replicates, the co-occurrence network results are more appropriate for reflecting potential ecological association patterns rather than confirmed microbial interactions, and the stability of the network structure as well as actual microbial interactions still require further validation.

In summary, under appropriate potassium application levels, plant growth is enhanced and beneficial microorganisms are enriched, whereas a high-potassium environment inhibits plant growth and may also increase the competitive advantage of potential pathogens, thereby amplifying ecological risks and adversely affecting cherry tomato growth. Therefore, excessive potassium application should be avoided in practical production. Instead, precise fertilization should be adopted to meet crop nutritional requirements while maintaining the synergistic stability of the plant–microbe system.

## 4. Materials and Methods

### 4.1. Plant Material and Experimental Design

The experiment was conducted in a solar greenhouse located in Hetian County, Xinjiang Uygur Autonomous Region, China (79°89′ E, 37°31′ N). The soil at the experimental site was classified as aeolian sandy soil. The basic physicochemical properties of the soil were as follows: pH 7.7, total nitrogen 0.049 g·kg^−1^, total phosphorus 0.90 g·kg^−1^, total potassium 18 g·kg^−1^, and available potassium 62 mg·kg^−1^. At the sampling stage, soil pH values under the K0–K4 treatments were 7.26, 7.44, 7.36, 7.32, and 7.35, respectively, indicating only minor variation among potassium treatments.

Cherry tomato (*Solanum lycopersicum* var. *cerasiforme*, cultivar ‘Tianshanhong’ (TSH)) seeds provided by the College of Agriculture, Shihezi University. Seeds were sown in 72-cell plug trays (cell volume ≈ 60 mL) filled with a seedling substrate consisting of peat, perlite, and vermiculite at a volume ratio of 2:1:1. Seedlings were raised in a controlled growth chamber (RXZ-300C, Ningbo Jiangnan Instrument Factory, Ningbo, China) under a day/night temperature regime of 26/18 °C, a photoperiod of 16 h light/8 h dark, a relative humidity maintained at 40–60%, and a light intensity of 300 μmol·m^−2^·s^−1^. When seedlings reached the five-leaf stage with a visible apical meristem, uniform and healthy seedlings were selected for transplanting. The experiment was conducted in the solar greenhouse. During the growing season, environmental parameters were recorded by an automatic monitoring system. The greenhouse air temperature ranged from 18.8 to 40.8 °C, and relative humidity ranged from 22% to 100%. Plants were grown under natural sunlight conditions, and the maximum midday canopy light intensity on sunny days was approximately 355 μmol·m^−2^·s^−1^.

**Figure 11:**
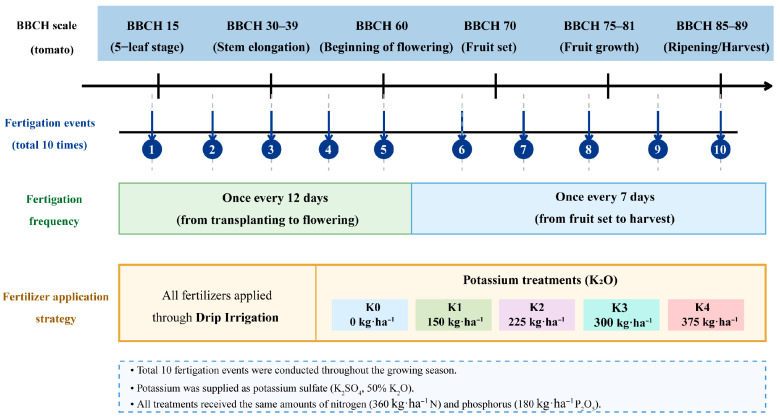
Schematic overview of the fertigation schedule, potassium application strategy, and corresponding developmental stages of cherry tomato plants based on the BBCH scale during the experimental period.

Potassium application (K0–K4) were designed based on local fertilization practices and agronomic recommendations for greenhouse cherry tomato production. Specifically, K3 (300 kg ha^−1^ K_2_O) represented the locally recommended potassium application. Five potassium application levels, expressed as K_2_O, were established: no potassium application (K0, 0 kg ha^−1^ K_2_O), low potassium (K1, 150 kg ha^−1^ K_2_O), moderate potassium (K2, 225 kg ha^−1^ K_2_O), conventional potassium (K3, 300 kg ha^−1^ K_2_O), and excessive potassium (K4, 375 kg ha^−1^ K_2_O). Potassium was supplied in the form of potassium sulfate (K_2_SO_4_, containing 50% K_2_O), with application rates per ridge for K0–K4 being 0 g, 321.68 g, 482.62 g, 642.60 g, and 803.16 g, respectively. The experiment followed a completely randomized design. Ridge width and spacing were both set at 40 cm. Each ridge was 14.85 m in length and 40 cm in width. For each of the five potassium treatments, three independent ridges were established as three biological replicates (*n* = 3). Each biological replicate (i.e., each ridge) initially contained 34 cherry tomato plants. At the sampling stage, five plants were randomly selected from within each ridge. The rhizosphere soil collected from these five plants was thoroughly mixed to form one composite sample per biological replicate. Consequently, for each potassium treatment, a total of three composite rhizosphere samples were obtained and subjected to DNA extraction and high-throughput sequencing. All treatments received the same amounts of nitrogen (360 kg·ha^−1^ N) and phosphorus (180 kg·ha^−1^ P_2_O_5_). Water-soluble diammonium phosphate (containing 18% N and 46% P_2_O_5_) was used as the phosphorus fertilizer, with each ridge receiving 298.18 g. Nitrogen fertilizer was provided through a combination of diammonium phosphate and urea (containing 46% N). After accounting for the nitrogen supplied by diammonium phosphate, an additional 784.38 g of urea was applied per ridge to ensure a consistent total nitrogen content across all treatments. All fertilizers were precisely applied through the drip irrigation system as part of an integrated water and fertilizer management approach. As shown in Figure 11, based on the dynamic nutrient patterns of cherry tomato plants at different growth stages, the total fertilizer amount was evenly distributed over 10 applications throughout the entire growing period. To better align with the nutrient demand dynamics of the tomato growth cycle, a locally adapted strategy for adjusting topdressing frequency was adopted: fertilization was applied once every 12 days during the early growth stage (from planting to flowering), and once every 7 days during the mid-to-late growth stage (from fruit set to harvest).

### 4.2. Soil Sample Collection

Cherry tomato seedlings were transplanted on 12 September 2024, and rhizosphere soil samples were collected at the fruit maturity stage on 21 December 2024. Within each replicate, five plants were randomly selected for rhizosphere sampling. Rhizosphere was collected using the root-shaking method. Briefly, plant root systems were carefully excavated, and loosely attached soil was gently shaken off. Soil tightly adhering to the root surface (approximately within 1–3 mm of the root surface) was then collected using a sterile brush. Samples from the same replicate were pooled to form one composite biological sample. All samples were immediately frozen in liquid nitrogen and subsequently stored at −80 °C until microbial DNA extraction.

### 4.3. Microbial DNA Extraction, PCR Amplification, and Sequencing

Total microbial genomic DNA was extracted from approximately 0.5 g of rhizosphere soil samples using the E.Z.N.A.^®^ Soil DNA Kit (Omega Bio-tek, Norcross, GA, USA), following the manufacturer’s instructions. DNA quality and concentration were assessed by 1% agarose gel electrophoresis and a NanoDrop 2000 spectrophotometer (Thermo Scientific, Waltham, MA, USA). Qualified DNA samples were used as templates for PCR amplification using barcoded, region-specific primers. For bacterial communities, the V3–V4 hypervariable region of the 16S rRNA gene was amplified using primers 338F (5′-ACTCCTACGGGAGGCAGCAG-3′) and 806R (5′-GGACTACHVGGGTWTCTAAT-3′). For fungal communities, the internal transcribed spacer 1 (ITS1) region was amplified using primers ITS1F (5′-CTTGGTCATTTAGAGGAAGTAA-3′) and ITS2 (5′-GCTGCGTTCTTCATCGATGC-3′). Each 20 μL PCR reaction contained 4 μL of 5× FastPfu buffer, 2 μL of dNTPs (2.5 mM), 0.8 μL of each primer (5 μM), 0.4 μL of FastPfu DNA polymerase, and approximately 10 ng of template DNA. The PCR program consisted of an initial denaturation at 95 °C for 3 min, followed by 27 cycles of denaturation at 95 °C for 30 s, annealing at 55 °C for 30 s, and extension at 72 °C for 30 s, with a final extension at 72 °C for 10 min.

PCR products were verified by 2% agarose gel electrophoresis, purified using a DNA gel extraction kit (Shanghai Majorbio Bio-Pharm Technology Co., Ltd., Shanghai, China), and quantified using a Qubit 4.0 fluorometer (Thermo Scientific, Waltham, MA, USA). Purified amplicons were used to construct sequencing libraries with the NEXTFLEX Rapid DNA-Seq Kit and sequenced on an Illumina NextSeq 2000 platform (Shanghai Majorbio Bio-Pharm Technology Co., Ltd., Shanghai, China) using paired-end 2 × 250 bp sequencing.

### 4.4. Determination of Plant Growth Phenotypes

At the fruit ripening stage, five uniformly growing plants were randomly selected from each plot for the measurement of morphological and dry matter traits.

Plant height was measured using a tape measure from the stem base to the main stem growth point. Stem diameter was measured at 1 cm above the cotyledon node using a digital vernier caliper (accuracy: 0.01 mm). For leaf area determination, five functional leaves were collected from the upper, middle, and lower positions of each plant, and the average single leaf area was measured using a portable leaf area meter (LI-3000C, LI-COR Biosciences, Lincoln, NE, USA).

To determine dry matter accumulation, each plant was divided into four organs: roots, stems, leaves, and fruits. All samples were rinsed with deionized water, then heat-treated in an oven at 105 °C for 30 min, followed by drying at 75 °C until a constant weight was achieved. The dry matter of each organ was weighed using a high-precision electronic balance (accuracy: 0.01 g). Total dry matter was calculated as the sum of the dry weights of all organs.

### 4.5. Bioinformatics and Statistical Analysis

For plant growth parameters and physiological traits (including plant height, stem diameter, leaf area, chlorophyll content, and dry matter accumulation), differences among treatments were determined by one-way analysis of variance (ANOVA), followed by Tukey’s Honestly Significant Difference (HSD) test for post hoc multiple comparisons at a significance level of *p* < 0.05.

Raw sequencing data were quality-filtered using fastp (v0.23.4) to remove low-quality reads. Paired-end reads were merged using FLASH (v1.2.11). Sequence clustering was performed using USEARCH (v11) at a 97% similarity threshold to generate operational taxonomic units (OTUs), and chimeric sequences were removed using the built-in chimera detection function. Taxonomic annotation was conducted using the RDP Classifier (v2.11) with a confidence threshold of 70%, against the SILVA 138 database for bacteria and the UNITE 9.0 database for fungi. Sequences annotated as chloroplasts or mitochondria (for bacteria) and non-fungal sequences (for ITS) were removed prior to downstream analyses. To minimize the effects of sequencing depth variation, all samples were rarefied to the minimum sequencing depth (46,778 reads per sample for bacteria and 30,000 reads per sample for fungi) for subsequent beta diversity analyses. Community analyses were performed using QIIME 2 (version 2022.11) and the “vegan” package in R (v4.2.2). Beta diversity was visualized by principal coordinates analysis (PCoA) based on Bray–Curtis distance matrices, and differences among groups were tested using permutational multivariate analysis of variance (PERMANOVA; Adonis, 999 permutations).

Differentially abundant microbial taxa among potassium treatments were identified using the online LEfSe (Linear Discriminant Analysis Effect Size) tool, with an LDA score threshold of 3.0. Microbial co-occurrence networks were constructed based on OTU-level relative abundance data using the SparCC algorithm (100 iterations). Strong correlations (|r| > 0.8, *p* < 0.05) were retained, and network visualization and topological properties (including number of nodes, number of edges, average degree, and modularity) were analyzed using Gephi (v0.10.1).

## 5. Conclusions

Potassium is an essential mineral nutrient in soils, and its availability is recognized as a key factor influencing soil microbial community structure and ecological functions. The present study demonstrates that gradient potassium application significantly affects rhizosphere microbial community composition and potential interaction networks through the modulation of cherry tomato root development. An appropriate potassium supply promoted plant growth, enhanced bacterial community diversity, and enriched nitrogen-fixing, biocontrol, and multifunctional metabolic taxa, with co-occurrence networks maintaining high connectivity and structural stability. In contrast, fungal communities exhibited a clear threshold response to potassium gradients. Core fungal groups collapsed under low-potassium stress, and the network underwent substantial reorganization under high-potassium conditions, with increased modularity accompanied by the enrichment of parasitic and potentially pathogenic groups. Overall, moderate potassium application (225 kg ha^−1^ K_2_O) appeared to provide the most favorable balance for cherry tomato growth and rhizosphere microbial stability, whereas excessive potassium input (375 kg ha^−1^ K_2_O) may increase the risk of fungal community imbalance and pathogen enrichment.

## Data Availability

The original contributions presented in this study are included in the article. Further inquiries can be directed to the corresponding authors.

## Appendix A

**Table A1:** Relative abundance (%) of dominant bacterial phyla in rhizosphere under different potassium treatments.

Phylum	K0	K1	K2	K3	K4
Pseudomonadota	29.19 ± 3.54 ^ab^	26.75 ± 0.54 ^b^	28.37 ± 1.61 ^ab^	27.14 ± 1.68 ^b^	32.59 ± 1.72 ^a^
Actinomycetota	18.20 ± 3.93 ^b^	24.27 ± 1.93 ^a^	7.80 ± 0.57 ^c^	8.25 ± 0.83 ^c^	8.15 ± 0.77 ^c^
Bacillota	9.79 ± 0.94 ^b^	14.92 ± 1.58 ^a^	16.94 ± 3.00 ^a^	17.85 ± 2.96 ^a^	15.54 ± 3.69 ^a^
Chloroflexota	11.55 ± 0.62 ^c^	19.74 ± 2.38 ^a^	18.32 ± 1.42 ^ab^	18.02 ± 1.54 ^ab^	18.28 ± 1.07 ^ab^
Acidobacteriota	4.17 ± 0.20 ^a^	4.30 ± 1.27 ^a^	5.20 ± 1.27 ^a^	4.17 ± 1.08 ^a^	4.47 ± 1.78 ^a^

Note: Differences were analyzed using one-way ANOVA followed by Tukey’s HSD test. Different lowercase letters within rows indicate significant differences at *p* < 0.05.

**Table A2:** Relative abundance (%) of dominant bacterial genus in rhizosphere under different potassium treatments.

Genus	K0	K1	K2	K3	K4
*Ensifer*	0.43 ± 0.01 ^c^	1.01 ± 0.24 ^b^	1.89 ± 0.34 ^a^	0.71 ± 0.21 ^bc^	0.72 ± 0.13 ^bc^
*Lysobacter*	0.08 ± 0.04 ^c^	0.15 ± 0.02 ^c^	1.04 ± 0.25 ^a^	0.17 ± 0.07 ^bc^	0.12 ± 0.02 ^bc^
*Gracilibacillus*	0.25 ± 0.05 ^c^	0.66 ± 0.10 ^b^	0.42 ± 0.12 ^bc^	1.76 ± 0.26 ^a^	0.46 ± 0.11 ^bc^
*Oceanobacillus*	0.48 ± 0.12 ^c^	0.98 ± 0.11 ^b^	1.30 ± 0.17 ^ab^	1.84 ± 0.36 ^a^	0.60 ± 0.05 ^c^
*Planifilum*	0.70 ± 0.03 ^c^	0.64 ± 0.04 ^c^	1.15 ± 0.27 ^b^	0.75 ± 0.17 ^c^	1.69 ± 0.16 ^a^

Note: Differences were analyzed using one-way ANOVA followed by Tukey’s HSD test. Different lowercase letters within rows indicate significant differences at *p* < 0.05.

**Table A3:** Topological parameters of bacterial co-occurrence sub-networks under different treatments.

Treatment	Nodes	Edges	Average Degree	Density	Average Path Length	Clustering Coefficient	Modularity
K0	621	917	2.953	0.00476	7.141	0.320	0.803
K1	627	927	2.957	0.00472	7.162	0.320	0.811
K2	625	943	3.018	0.00484	7.178	0.321	0.795
K3	628	916	2.917	0.00465	7.429	0.316	0.810
K4	628	934	2.975	0.00474	7.208	0.321	0.800

**Table A4:** Topological parameters of fungal co-occurrence sub-networks under different treatments.

Treatment	Nodes	Edges	Average Degree	Density	Average Path Length	Clustering Coefficient	Modularity
K0	83	91	2.193	0.02674	3.361	0.405	0.730
K1	82	87	2.122	0.02620	4.270	0.373	0.785
K2	83	90	2.169	0.02645	3.163	0.433	0.795
K3	90	124	2.756	0.03096	3.194	0.455	0.766
K4	99	126	2.545	0.02597	3.794	0.571	0.829
